# Measurement effects on critical scaling in neural systems

**DOI:** 10.3389/fncom.2025.1724190

**Published:** 2026-01-23

**Authors:** M. Shane Li, Benyuan Liu, Keith W. van Antwerp, Eslam Abdelaleem, Audrey J. Sederberg

**Affiliations:** 1 School of Physics, Georgia Institute of Technology, Atlanta, GA, United States; 2 School of Psychology, Georgia Institute of Technology, Atlanta, GA, United States

**Keywords:** calcium imaging, coarse graining, electrophysiology, neural criticality, population dynamics, renormalization group

## Abstract

The recently developed phenomenological renormalization group (pRG) analysis has uncovered scale-free properties in large-scale neural population recordings across recording modalities, including extracellular electrophysiology and calcium imaging. The convergence of these properties across the datasets hints at universal neural behavior. Yet, it is unknown how differences in temporal resolution and measurement details affect pRG scaling. Here, we use a network model known to produce scaling under pRG analysis as a testbed to assess how recording and analysis choices shape inferred scaling exponents. We show that scaling properties depend on the choices of temporal binning, measurement nonlinearities, and deconvolution, and that the quality of scaling for cluster covariance eigenvalues is particularly sensitive to measurement effects. Moreover, all scaling exponents shift substantially with these transformations, even when the underlying neural dynamics are identical. Together, these results show how experimental choices can change pRG scaling and provide a framework for separating scaling driven by neural dynamics from that introduced by the recording method.

## 1 Introduction

Criticality is a leading candidate framework for understanding brain function and dysfunction (Chialvo, 2010; Hengen and Shew, 2025), potentially accounting for what dynamical states are optimal for learning and computation (Legenstein and Maass, 2007; Kinouchi and Copelli, 2006; Shew and Plenz, 2013; Safavi et al., 2024; Cambrainha et al., 2025) and what goes awry in disease (Rocha et al., 2022; Xu et al., 2024; McGregor et al., 2024). Over the past few decades, criticality has been extensively explored in neural data, including spiking activity in small populations, LFP, and M/EEG (Beggs and Plenz, 2003; Petermann et al., 2009; Shew et al., 2015; Fontenele et al., 2019; Ponce-Alvarez et al., 2018), and much work has examined the challenges of interpreting such analyses (Touboul and Destexhe, 2017; Aitchison et al., 2016; Priesemann and Shriki, 2018; Levina et al., 2022; Morrell et al., 2024). Neural populations numbering in the thousands are now routinely recorded by modern imaging and extracellular electrophysiology (Stringer et al., 2019; Chung et al., 2019; Steinmetz et al., 2021; Demas et al., 2021; Angelaki et al., 2025), and new datasets open new approaches to the study of criticality. While these high-dimensional datasets are complex, insight from statistical physics suggests that not all details matter: the renormalization group (RG) shows us that microscopic details can be irrelevant to macroscopic behavior. In this spirit, a phenomenological renormalization group (pRG) approach was recently adapted from statistical physics to neural data (Meshulam et al., 2019, 2023). This analysis systematically coarse-grains population activity to reveal scale-invariant relationships across levels of description. The pRG analysis has now been applied across multiple recording modalities, including electrophysiology across brain areas (Morales et al., 2023), cortical states (Castro et al., 2024), and behavioral tasks (Cambrainha et al., 2025), as well as calcium imaging in diverse species (Munn et al., 2024) and fMRI in humans (Calvo et al., 2025). However, even when robust scaling is observed, the meaning of scaling exponents, and their interpretation across diverse systems and recording methods, remain uncertain. In particular, it is unknown which details of experimental design and measurement matter for pRG analysis.

The first work on RG-based approaches to the analysis of large neural populations was based on recordings of 2000+ neurons in the mouse hippocampus using two-photon calcium imaging (Meshulam et al., 2019). Briefly, their analysis proceeds by iteratively clustering neurons into groups based on activity correlations and assessing cluster activity distributions, variance, covariance matrix spectrum, and autocorrelation across scales. When scaling of these quantities is observed, the outcome of this analysis is a set of scaling exponents that reflect the spatial and temporal correlation structure of the system. Meshulam et al. (2019) showed that several statistical and dynamical properties of neural activity collapsed onto the same forms, which were invariant across two orders of magnitude in scale.

The pRG analyses have now been applied across diverse neural systems and recording modalities, and in several cases the resulting scaling exponents appear surprisingly consistent. For example, Munn et al. (2024) examined PRG scaling across five different species, using recordings from very different experimental set-ups, and found nearly identical scaling of variance. Additionally, similar values of scaling exponents were reported in mouse hippocampus using two-photon calcium imaging (Meshulam et al., 2019) and later using high-density extracellular electrophysiology (Morales et al., 2023), despite substantial differences in temporal resolution, processing pipelines, and measurement physics. Each of these factors could influence the estimated exponents. Understanding the extent to which such observational factors shape pRG scaling is essential for determining when comparisons across modalities, species, or experiments reflect underlying neural dynamics rather than differences in data acquisition or processing. Recent work has begun to explore aspects of this issue, including supplemental analyses in multiple studies examining how exponents vary with temporal coarse-graining (Morales et al., 2023; Castro et al., 2024), though these results are not yet consistent across datasets. Together, these considerations underscore the need for a systematic framework to characterize how pRG outcomes depend on the transformations introduced by measurement and processing.

Directly addressing these questions with experimental data is challenging, as it would require simultaneously recording large populations of neurons with both electrophysiology and imaging. Instead, here we use a model of spiking activity whose pRG scaling properties match those observed experimentally (Morrell et al., 2021). Our aim is to examine how observational transformations affect the inferred exponents. We instantiate a experimentally tuned forward model using GCaMP6f-like kinetics (Wei et al., 2020) and apply commonly used out-of-the-box deconvolution tools (Giovannucci et al., 2019). With these controlled conditions, we examine three classes of observational transformations:

1. Temporal resolution: how the choice of spike-train bin size shapes the apparent existence and quality of scaling.
2. Nonlinear measurement dynamics: how the fluorescence transients associated with calcium indicators alter scaling exponents.
3. Deconvolution and processing: whether “raw” pRG exponents are recoverable from standard imaging pipelines.

In the remainder of the paper, we first provide an overview of the pRG analysis and our modeling testbed. We then examine how the scaling of variance (α), free energy (β), and covariance structure (μ) are modulated by observational transformations. We observe that scaling quality is maintained for variance and free energy, but degrades for the covariance structure, and we find changes in all exponents with these transformations. These results establish that observational transformations can differentially impact the exponents recovered by pRG analyses. Taken together, this work provides a framework that can be used to distinguish scaling signatures that reflect neural dynamics from those arising from experimental and analytical constraints, and for clarifying how methodological choices impact efforts to identify criticality in neural systems.

## 2 Scaling and temporal binning

### 2.1 Overview of the pRG analysis

Meshulam et al. (2019) introduced the pRG procedure to quantify the self similarity of the system across scales. In the “real space” version of the pRG analysis, neurons are iteratively clustered based on maximum pairwise correlations, and properties of these clusters are tracked across scales (see Section 5.2). Several properties scale with the cluster size: the variance of cluster activity ($\sigma_{k}^{2}\sim K^{\alpha }$), the “free energy” of the cluster ($F=-\ln\;P_{\text{silence}}\sim K^{-\beta }$), the eigenvalues of the cluster covariance (λ(*r*) ~ (*r*/*K*)^−μ^), and the time constant of cluster activity autocorrelation ($\tau_{c}\sim K^{\tilde{z}}$). Here, we focus on the three “stationary” scaling relations (α, β, μ). The exponents characterizing these relationships depend on the correlation structure within the population, and they deviate from the trivial scaling relationship expected in any independent system (i.e., α = 1, β = 1, μ = 0). The fourth exponent, $\tilde{z}$, is dynamical in nature and depends on the intrinsic time constants of the activity, and we discuss this in more detail in Section 4.

Cross-correlations between neurons computed from spike-counts are well-known to depend on the spike count bin size (Δ) (Kass and Ventura, 2006; Cohen and Kohn, 2011). Throughout the pRG analysis, correlations play a large role: they are used to determine coarse-grained clusters, and one of the scaling relationships observed derives from the eigenvalue spectrum of the cluster covariance matrix. Thus, at multiple points in the coarse-graining analysis, time bin size has a role. In this section, we first introduce a network model with known scaling behavior (Morrell et al., 2021) and then examine the sensitivity of the coarse-graining analysis to the choice of temporal binning.

### 2.2 A model testbed for scaling behavior

The model has been described in detail elsewhere (Morrell et al., 2021, 2024). Briefly, individual neurons are modeled as binary units for which the probability of firing in any time bin is determined by the activation of *N*_*F*_ latent fields, weighted by the coupling $J_{im}\sim N\left(0,N_{F}^{-1}\right)$ between the neuron *i* and each of the fields *m*. Each latent field is modeled as a mean-reverting random walk with autocorrelation time τ_*F*_. The overall strength of coupling to latent fields is controlled by the parameter η (“coupling”). A global bias ϵ adjusts the average population firing rate. We simulated activity in *N* = 2^10^ neurons with η = 3, ϵ = 8 with *N*_*F*_ = 10 fields, equivalent to the parameters used in Morrell et al. (2021) that produced good scaling (Table 1; see Supplementary Figure 1).

**Table 1:** Dynamic latent variable model parameters.

**Parameter**	**Description**	**Value**
ϵ	Bias toward silence	8
η	Variance multiplier	3
*N*_*F*_	Number of latent fields	10
τ_*F*_	Latent field time constant (s)	1.0
*dt*	Simulation step size (s)	0.01
Δ	Spike count bin size (s)	[0.01, 10.0]
*T*	Total simulation length (s)	20, 000
*J*_*im*_	coupling between neuron *i* and field *m*	$\sim N\left(0,N_{F}^{-1}\right)$
*K*	cluster size in coarse-graining	32, 64, 128, 256
μ	covariance eigenvalue scaling exponent	(0.7, 1.4)
α	variance scaling exponent	(1.2, 1.5)
β	variance scaling exponent	(0.8, 1.0)

**Figure 1:**
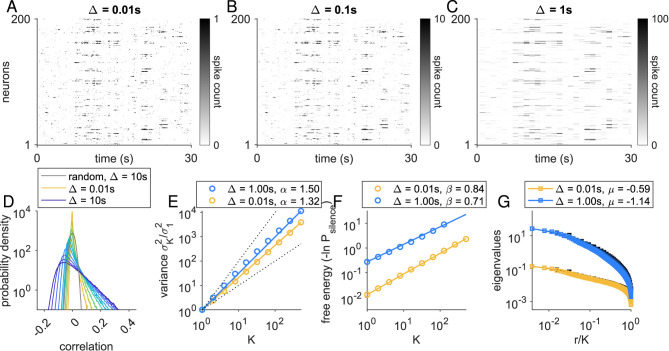
Coarse-graining analysis results depend on temporal binning. **(A–C)** Illustration of temporal binning, for Δ = 0.01, 0.1, 1 s, on a 30-s sample of activity in 200 neurons simulated using the dynamic latent variable model (details, Section 5.1). **(D)** Distribution of correlations for simulated data with time bins ranging from Δ = 0.01 s (orange) to Δ = 10 s (dark blue). Gray: distribution of correlations for Gaussian random data with number of samples matched to the full simulation binned at Δ = 10 s. **(E–G)** pRG analysis (details, Section 5.2) results for Δ = 0.01 s and Δ = 1 s. **(E)** Cluster variance vs. cluster size, on log-log scale. **(F)** Free energy (defined as the negative natural logarithm of the probability of silence in a cluster) vs. cluster size, on log-log scale. **(G)** Eigenvalues vs. normalized rank, on log-log scale.

Because the model yields the probability of activity in each time step (see Section 5.1, Equation 1), setting the step size *dt* fixes the range of firing rates in the network. We set the simulation step size *dt* = 0.01 s, and so the distribution of neural firing rates ranged from 0.1 to 10 Hz. For simplicity, all latent fields have the same time constant (τ_*F*_ = 1 s). We defined the spike count bin to be Δ, and this ranged from 0.01 s (= *dt*) to 10 s. A sample of binned spike counts from 200 neurons over a 30 s period are shown for bin sizes Δ = 0.01, 0.1, 1 s in Figures 1A–C. The total simulation length was *T* = 20, 000 s (Section 5.1; see Supplementary Figure 2 for finite-length analysis).

We examined the distribution of correlations as Δ was increased (Figure 1D). While coupling in the model is random, the correlations are always much larger than expected from randomized data matched to simulation length (gray curve, Figure 1D). When the Δ ≪ τ_*F*_, correlations are determined by the coupling *J*_*im*_ to the latent field *h*_*m*_(*t*), but correlations are small because spiking is probabilistic and spike rates are low. As Δ increases, noise in the spike generation is averaged out, and correlations increase. Thus, we expect that as Δ increases, exponents will move away from the random (independent) model values: α and μ will increase, while β will decrease.

### 2.3 Loss of scaling quality with larger time bins

When coarse graining was performed at the simulation resolution (Figure 1A), we found clear scaling in the cluster variance (Figure 1E, orange), free energy (Figure 1, orange), and eigenvalue spectrum (Figure 1D, orange). Using the same simulation, we re-binned the spiking activity with Δ = 1 s and repeated the coarse graining analysis. Qualitatively, scaling was preserved, but scaling relationship specifics changed (blue lines, Figures 1E–G). As expected, α and μ increased, while β decreased.

**Figure 2:**
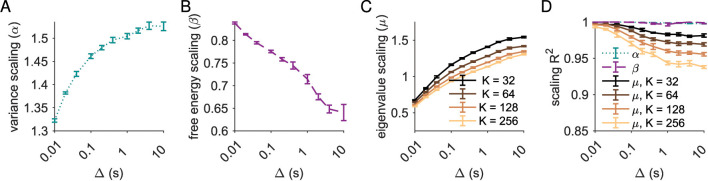
Scaling exponents depend on temporal binning. **(A)** Variance scaling exponent α increases with bin size Δ. **(B)** Free energy scaling exponent β decreases with bin size Δ. **(C)** Eigenvalue scaling exponent μ increases with bin size Δ. **(D)** Quality of scaling relationship for variance (α, teal) and free energy (β, plum) is high regardless of bin size while eigenvalue scaling quality degrades with larger bin sizes (solid lines). Error bars are ±1s.d. across fifths of the simulation.

To examine this in greater detail, we next examined the scaling relationships and quality of scaling as a function of bin size Δ (Figure 2). Quality of scaling was assessed by *R*^2^ of the linear fit in log-log space computed over log-spaced points (see Section 5.2) and, for eigenvalue scaling, by examining the spread in slope (μ) across cluster sizes *K* (Figure 2D). At the simulation resolution (Δ = *dt* = 0.01 s), the quality of scaling of the variance with the cluster size (σ^2^ ~ *K*^α^ ) was very high (*R*^2^ > 0.999), with scaling exponent α = 1.323 ± 0.003. As Δ increased, variance still scaled with cluster size (*R*^2^ > 0.999), but the scaling exponent increased to α = 1.51 ± 0.01 (Figures 2A, D). Similarly, the quality of free energy scaling remained high (*R*^2^ > 0.99) as Δ increased (Figure 2D). At simulation resolution we found scaling exponent β = 0.838 ± 0.002, and at the maximum Δ = 10 s we found β = 0.714 ± 0.010 (Figure 2B).

At the simulation timescale (minimum Δ = *dt*), the quality of eigenvalue scaling was high (*R*^2^ > 0.99 across all *K*) and values of μ changed little with *K*, ranging from μ = 0.66 ± 0.02 at *K* = 32 to μ = 0.59 ± 0.01 at *K* = 256. As Δ increased, the quality of eigenvalue scaling degraded, with *R*^2^ = 0.98 for *K* = 32 decreasing to *R*^2^ = 0.94 for *K* = 256, and increasing spread with cluster size *K* (Figure 2D). For instance, at Δ = 1 s, the scaling exponent μ ranged from μ = 1.41 ± 0.01 for *K* = 32 to μ = 1.13 ± 0.01 for *K* = 256. While the overall range across *K* for μ was only 0.07 for Δ = 0.01 s, it was four times larger (0.28) for Δ = 1 s (Figure 2C), suggesting a breakdown in the quality of scaling.

In summary, variance and free energy scaling is very robust: the quality of scaling is high at all bin sizes. By contrast, increasing bin size degrades the quality of scaling for eigenvalue vs. rank. In all cases, the value of the scaling exponent depends strongly on bin size.

## 3 Scaling in simulated imaged populations

The pRG exponents reported from imaged activity (Meshulam et al., 2019) and electrophysiologically recorded activity (Morales et al., 2023) in the hippocampus were similar, within small experimental error bars. Given the results of the previous section that exponents depend on the temporal resolution of recorded activity, this similarity is surprising, the more so because temporal resolution is only one difference between imaging and electrophysiology. Usually, neural activity is inferred by deconvolving imaged fluorescence, which reflects calcium dynamics as well as the indicator response function, both of which are heterogeneous across cells. Thus, having established that temporal coarse-graining alone can distort apparent scaling exponents, we next asked what distortions arise from the calcium-imaging process itself. In this section, we implement a forward model for imaged calcium dynamics and apply a standard deconvolution algorithm to infer spiking activity. We then compare the results of a coarse-graining analysis applied to spiking activity, simulated imaged calcium, and deconvolved activity.

### 3.1 Simulation: calcium dynamics, fluourescence, and deconvolution

**Figure 3:**
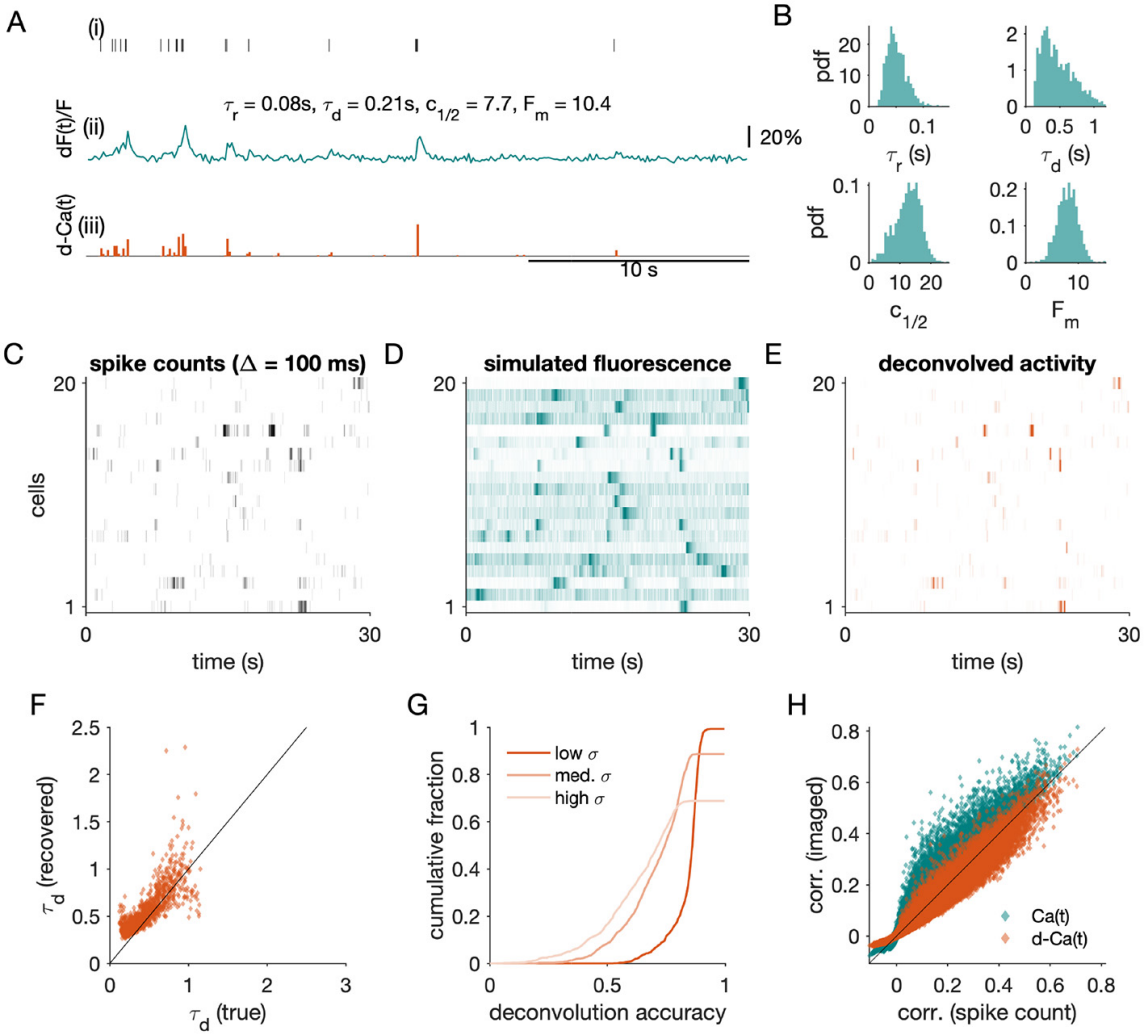
Forward model for calcium imaging and recovery of population activity. **(A)** Example single cell showing (i) simulated spike train, (ii) synthetic fluorescence generated using a GCaMP6f-like forward model, and (iii) deconvolved activity obtained with the constrained foopsi algorithm (Giovannucci et al., 2019). Spikes are simulated at 10 ms resolution and fluorescence is sampled at 10 Hz (Δ = 100 ms). **(B)** Distributions of calcium-model parameters across the population (τ_*r*_, τ_*d*_, *c*_1/2_, *F*_*m*_), chosen to approximate those reported for GCaMP6f-TG (Wei et al., 2020). **(C–E)** Population activity for 20 example cells over a 30 s window: **(C)** binned spike counts, **(D)** simulated fluorescence traces, and **(E)** deconvolved activity. In **(D)**, each row is normalized by its range for visibility. **(F)** Recovered decay time constants are strongly correlated with ground-truth values in the low-noise condition. **(G)** Deconvolution accuracy (correlation between inferred and true spike counts) declines systematically with increasing external noise. **(H)** Pairwise correlations from fluorescence (Ca(*t*)) and deconvolved activity (d-Ca(*t*)) closely track spike-count correlations, with smaller deviations after deconvolution.

**Figure 4:**
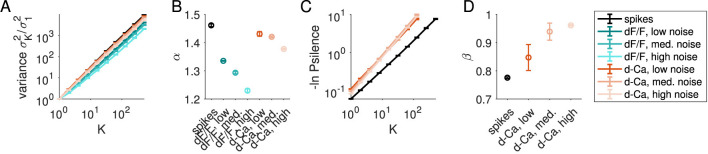
Variance and free-energy scaling exponents derived from spikes, simulated fluorescence, and deconvolved calcium signals. **(A)** Variance scales with cluster size as *K*^α^ for spike counts (Δ = 100 ms), simulated fluorescence traces (*dF*/*F*) at three noise levels, and deconvolved fluorescence traces (*d*−*Ca*(*t*)). All conditions exhibit a clear power-law relationship. **(B)** The exponent α decreases with increasing external noise in the fluorescence simulation. Deconvolution partially restores α toward the spike-based value. **(C)** Free-energy, quantified by −ln *P*_silence_, scales with cluster size as *K*^β^ for spike counts and deconvolved fluorescence traces. **(D)** The exponent β is higher in the deconvolved signal and increases with added noise. Because the simulated fluorescence traces include baseline fluctuations and lack a true zero-activity state, β cannot be defined reliably for raw *dF*/*F*; we therefore report β only for spike-count and deconvolved data. Error bars denote ±1 s.d. across fifths of the data.

Obtaining simultaneous large-scale electrophysiology and calcium imaging from the same neuronal population is not currently feasible. However, paired electrophysiology-imaging recordings at the single-cell level constrain forward models from spikes to fluorescence (Wei et al., 2020), enabling a controlled simulation-based approach to examine how calcium indicator dynamics and noise influence the pRG exponents. Starting from the simulated spiking activity analyzed in Section 2 (single cell in Figure 3Ai; population subset, Figure 3C), we generated fluorescence traces using the forward model of Wei et al. (2020) and sampled the resulting signals at 10 Hz (Δ = 100 ms), matching typical imaging frame rates (Figure 3Bii; population, Figure 3D). To set model parameters (τ_*d*_, τ_*r*_, *F*_*max*_, *c*_1/2_, *k*) across the population (Figure 3B, Table 2), we approximated distributions shown for GCamp6f-TG (Wei et al., 2020; see Figure 4 therein). We deliberately include this heterogeneity in model parameters to reflect heterogeneity observed across real populations of neurons. We simulated fluorescence traces with three levels of external noise, σ_ext_ = 0.02, 0.2, and 0.4.

**Table 2:** Simulated calcium imaging parameters.

**Parameter**	**Description**	**Value**
τ_*d*_	calcium decay time (s)	≈0.5
τ_*r*_	calcium rise time (s)	≈0.05
*F*_*max*_	maximum fluorescence	≈8 ± 2
*c*_1/2_	calcium to fluorescence threshold	~5
*k*	sharpness	~2
σ_*ext*_	external noise	{0.02, 0.2, 0.4}

We then deconvolved the simulated fluorescence using standard methods (Giovannucci et al., 2019), producing inferred spike trains for each neuron (Figure 3Aiii; population, Figure 3E). Calcium decay time constants were inferred from simulated traces using short segments of data (10 segments of 100 s), and we confirmed that recovered time constants were close to the ground-truth values for most cells (Figure 3F), with larger errors for some cells with τ_*d*_ ≈ 1 s. The latent dynamics of the population model have an autocorrelation time τ_*F*_ = 1 s, and these errors may arise from cells that are strongly coupled to the latent dynamics.

In our our controlled, simulation-based environment, deconvolution was highly accurate, but decreased at higher noise levels. For the low-noise simulation, deconvolution ran successfully for 1,017 of 1,024 cells, and the median accuracy, quantified by the correlation between ground-truth spike counts and the deconvolved signal, was 0.86 (inter-quartile range (IQR), 0.05) (Figure 3E). Accuracy degraded with increasing noise level, with fewer activity traces successfully deconvolved (medium noise: 908 of 1024 successful; high noise: 705 of 1024 successful) and lower accuracy following successful deconvolution (medium noise: 0.72 (IQR, 0.18); high noise: 0.63 (IQR, 0.22)).

Using our simulations, we next asked how the transformations from spiking activity to fluorescence to deconvolved activity affected the cell-cell correlations that form the basis of the pRG coarse-graining (Figure 3H). Correlations were highly preserved: Spearman's ρ was 0.98 between the spike-count and simulated fluorescence correlations, and likewise 0.98 between spike-count and deconvolved-trace correlations. Correlation magnitudes were slightly increased in the simulated fluorescence (mean change +0.004 ± 0.018 relative to spike counts) and slightly decreased after deconvolution (−0.002 ± 0.019 relative to spike counts).

For fluorescence traces, we analyzed scaling relationships at the native imaging resolution (Δ = 100 ms). Unlike spike counts, where temporal coarse-graining can be performed over sub-millisecond resolution, calcium imaging already imposes a coarse temporal sampling interval, and additional binning would substantially reduce the number of independent time samples. Consistent with previous pRG analyses of calcium imaging (Meshulam et al., 2019; Munn et al., 2024), we therefore analyzed the fluorescence traces at a single value of Δ.

### 3.2 Scaling of variance and free-energy in simulated imaged activity

As in the earlier analyses, activity was coarse-grained into clusters of size *K*, now for each of seven conditions: spike counts (Δ = 0.1 s), simulated fluorescence traces (*dF*/*F*, for low, medium, and high noise) and deconvolved fluorescence traces (*d*−*Ca*(*t*)) at the same noise levels. Variance scaling fit quality remained high (*R*^2^ > 0.99) across all levels of noise, for both simulated fluorescence and the deconvolved signals (Figures 4A, B). Increasing noise levels decreases α, following expectations: fully uncorrelated data would show α = 1. We found the scaling exponent α was substantially lower for the simulated fluorescence (α = 1.334 ± 0.004 for the low-noise case) than for spike counts (α = 1.46 ± 0.01) or for deconvolved spike counts (α = 1.43 ± 0.01). One reason for this may be that the simulated fluorescence reflects the timing of the activity within the spike count window Δ, which adds variability to the signal that is not present in the summed spike counts in a window of the same size, thereby decreasing α. Deconvolution, which infers the spike count, appears to largely remove this variability, as α is similar between spike count and deconvolved data. This suggests that variance scaling can be recovered across multiple recording modalities.

We also computed the free-energy scaling exponent β (Figures 4C, D). Because the simulated fluorescence includes baseline noise and lacks a true zero-activity state, β cannot be reliably defined for the raw *dF*/*F* signals, and we therefore report β only for the spike-count and deconvolved data. As for α, the fit quality remained high for all conditions (*R*^2^ > 0.99). Increasing noise levels increases β: noise contributes spurious non-zero activity, which causes the population silence probability to decrease faster (i.e., higher β) with increasing cluster size than it would otherwise. These results indicate that both α and β are less sensitive to transformations: the quality of scaling fits remains high, while the exponents change predictably with increasing levels of noise.

### 3.3 Scaling of covariance eigenvalues in simulated imaged activity

**Figure 5:**
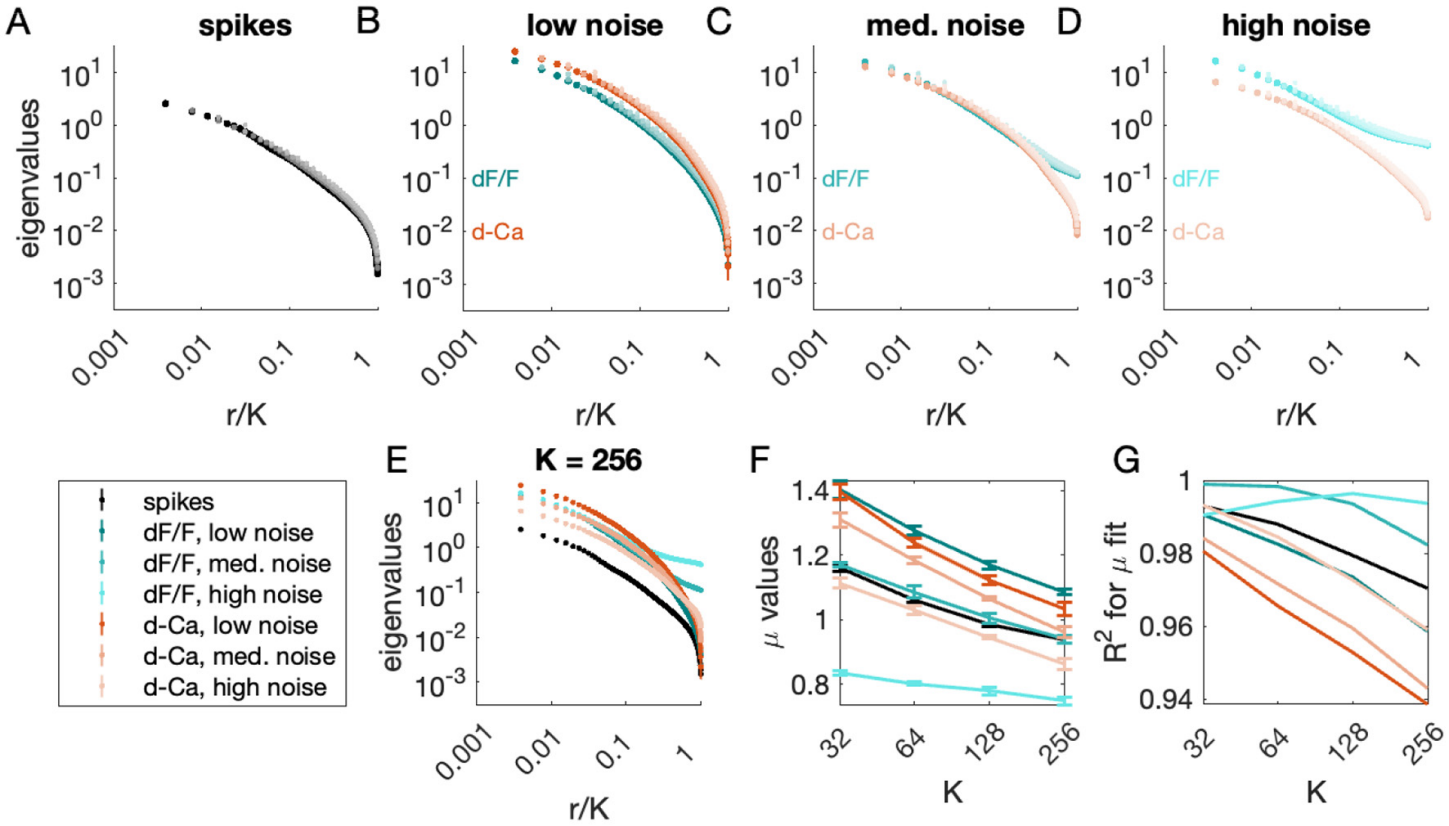
Comparison of eigenvalue scaling exponents derived from spike counts, calcium traces, and deconvolved data (Δ = 100 ms). **(A–D)** Eigenvalues vs. rank, for clusters of size 32, 64, 128 and 256 for **(A)** the spike counts, and the calcium traces (teal) and deconvolved data (red-orange) for **(B)** low noise, **(C)** medium noise, and **(D)** high noise conditions (see Section 5.3). **(E)** Eigenvalues vs. rank, for clusters of size *K* = 256 across different experimental and noise conditions. Colors as in **(A–D)**. **(F)** Eigenvalue scaling exponent μ vs. *K* for each signal. **(G)** *R*^2^ of fit to power-law decay (see Section 5.2) for each signal at each cluster size. Error bars on α and μ are the standard deviation across fifths of the data.

Next, we examined how eigenvalue scaling transformed, from the covariance obtained from spike counts (Δ = 0.1 s, Figure 5A), imaged fluorescence (Figures 5B–D, teal), and deconvolved fluorescence (Figures 5B–D, red, ‘d-Ca') at low (B), medium (C), and high (D) noise levels. In each case, the plot of cluster eigenvalues against scaled rank collapsed onto a curve across cluster sizes *K* = 32, 64, 128, 256. Figure 5E shows this curve for the *K* = 256 cluster eigenvalues across conditions. We quantified the eigenvalue scaling by extracting μ for each condition at each cluster size. As observed previously, μ varies somewhat with *K*, even for spike counts.

At very low noise levels (darkest red/teal lines, Figure 5F), the calcium dynamics shift μ from the spike-count value significantly: at all values of *K*, there is a positive shift in μ of 0.15 to 0.2 from spiking to simulated fluorescence. Deconvolution decreases this shift slightly, but does not recover the scaling exponents obtained from spike counts directly.

Deconvolution had a larger impact on exponents under noisier conditions (medium, high noise cases; light red/teal lines), and as expected, increasing noise in the simulated fluorescence decreased μ. The spectra plotted in Figures 5C–E suggest that the reason for this decrease in μ is that the white noise adds an offset across all eigenvalues, flattening the eigenvalue vs. rank curve. The effect of deconvolution on noisy calcium traces is to increase μ, by 0.02 to 0.14 for medium noise (σ = 0.2) and 0.11 to 0.28 for high noise (σ = 0.4).

Finally, we examined fit quality across conditions. The quality of fit assessed by *R*^2^ was comparable to that found in experimental observations (> 0.95) (Figure 5G). However, the highest quality fits were observed in noisy simulated calcium traces (teal curves, Figure 5G), largely due to the flattening of the curve in the tail. Fit quality was lower for deconvolved signals (red curves, Figure 5G) relative to simulated calcium traces, but still was higher for higher noise than lower noise. Thus, it is possible for a white-noise background to inflate fit quality for the eigenvalue scaling observed under pRG analysis. Moreover, the scaling exponent is highly sensitive to noise level and method of recording.

## 4 Discussion

The phenomenological renormalization group (pRG) offers a novel approach to probing criticality in large-scale neural recordings (Meshulam et al., 2019), emphasizing how population statistics transform across coarse-grained scales rather than relying on avalanche detection or reduction to a one-dimensional activity signal, which can obscure signatures of criticality (Dahmen et al., 2019; Fontenele et al., 2025). Given this promise, which is also reflected in its rapid adoption across electrophysiology, calcium imaging, and fMRI datasets (Morales et al., 2023; Castro et al., 2024; Munn et al., 2024; Calvo et al., 2025), it becomes increasingly important to understand the methodological limits of pRG analyses. In particular, comparisons across modalities require understanding how temporal sampling, nonlinear measurement dynamics, and processing choices shape the scaling relationships used to infer criticality. In this work, we examined how temporal coarse-graining and the nonlinear transformations inherent in calcium imaging and deconvolution impact the scaling relationships extracted via pRG, and we found that both factors can substantially alter the apparent scaling exponents.

### 4.1 Trends for each exponent

For overall context, trivial scaling (α = 1, β = 1, μ = 0) is observed in a system of independent variables, and if scaling persists with increasing levels of correlational structure, these exponents shift systematically: α and μ increase with correlation and β decreases. The trends we observed broadly reflect this intuition: more noise drove the system closer to randomness while averaging over this noise had the opposite effect.

#### 4.1.1 Variance scaling (α)

None of the forward-modeling steps substantially degraded the quality of the variance-cluster size relationship, and in our controlled setting, the exponent α changed in predictable ways with added noise or temporal filtering. For example, α decreased when external noise is increased and increased when noise is reduced, such as when we used larger time bins or after deconvolution. The trend with bin size was consistent with recent supplemental analyses (Morales et al., 2023, Figure S6; Castro et al., 2024, Figure S1). The most substantial departure arose when moving from spike counts to raw fluorescence: the simulated fluorescence introduced additional temporal variability tied to precise spike timing, which is not present when spikes are directly summed within a bin. This additional variability lowered the apparent α. Deconvolution appeared to remove much of this variability, with scaling exponents approaching those obtained from spike counts directly.

#### 4.1.2 Free energy scaling (β)

Similar to variance scaling, the quality of free energy scaling remained high under the transformations examined here. When noise was reduced via larger time bins, β decreased, which is also in agreement with recent results (Morales et al., 2023, Figure S6; Castro et al., 2024, Figure S1). We did not calculate β for simulated fluorescence, because free energy calculations require defining the zero-activity state (silence), which would have required an additional thresholding step on the simulated fluorescence, which is at any rate accomplished in the deconvolution step. The addition of high levels of noise in the simulated imaging pipeline drove β toward 1 (trivial scaling, independent neurons), as deconvolution had limited success at high noise levels.

#### 4.1.3 Eigenvalue scaling (μ)

Eigenvalue scaling was more sensitive than variance or free energy scaling to temporal binning, measurement nonlinearities, and deconvolution. This sensitivity appeared in two forms: the inferred exponent (μ) shifted systematically with noise and the quality of scaling fits changed with these transformations. Similar to variance scaling trends, μ increased when noise levels decreased. This was seen in the increase of μ with bin size Δ and in the decrease in μ with increasing external noise, from μ ≈ 1.4 under low noise to μ ≈ 0.8 under high noise. The dependence of μ on bin size has been examined previously in supplementary analyses supporting other studies (Morales et al., 2023; Castro et al., 2024), but the trends reported therein were discrepant with each other. Whether this discrepancy arises from differences in underlying dynamics [Castro et al. (2024) recorded only from the visual cortex while Morales et al. (2023) compiled recordings from across many brain areas], or a difference in processing is not clear, but our results were consistent with the supplementary analysis found by Morales et al. (2023) (see Figure S6).

One counterintuitive finding was that apparent scaling *quality* (as measured by *R*^2^ or collapse across cluster sizes) could improve when noise was added to the simulated fluorescence. White noise introduces a flat component across all covariance modes, masking deviations from a power-law tail and yielding apparently better scaling.

#### 4.1.4 Future work: dynamical scaling ($\tilde{z}$)

The pRG framework can also be applied to temporal structure by examining the scaling of autocorrelation times τ_*c*_ of cluster activity, yielding a dynamic exponent $\tilde{z}$ (Meshulam et al., 2019). In the context of the dynamic latent variable model, dynamic scaling behavior depends on the intrinsic time constants of the underlying latent variables (see, e.g., Figure S7 ( C ) of Morrell et al. (2021)). Depending on the choice of time constants in the model, the quality of dynamical scaling is often substantially lower than that of the static exponents α, β, and μ. In the present work, we introduced additional temporal structure through temporal binning, calcium indicator dynamics, and deconvolution. Future work incorporating $\tilde{z}$ will require exploring the space of intrinsic and observational time constants.

In summary, while α, β and μ ideally reflect intrinsic properties of the underlying neural dynamics, they differ in their susceptibility to measurement effects. Under identical conditions, μ, which captures structure across the full covariance eigenvalue spectrum, was more sensitive to observation noise and nonlinearities, whereas α and β were easier to measure. Even when the quality of scaling remained high, the size of the shifts we observed were on the order of the variations in exponents seen across brain areas (Morales et al., 2023). Overall, our results highlight how measurement effects can drive large differences in apparent scaling exponents.

### 4.2 Critical dynamics and the dynamic latent variable model

Our finding that α increases with time bin size (Δ) aligns with recent modeling work examining how temporal coarse-graining shifts α in a critically tuned random recurrent network model (Calvo et al., 2025). Of note, the exponent ranges for α (1.2 to 1.5) in a network with symmetric connections are similar to those found here. The model we use is, on the surface, quite different from a critically tuned dynamical network. However, under some circumstances, they may be equivalent, much in the same way that a restricted Boltzmann machine, with hidden variables controlling observed patterns, can approximate a Hopfield network, in which attractors arise from interactions (Smart and Zilman, 2021). In this view, our statistical network model, where population patterns are assumed to arise from coupling to a few latent variables, could be mapped onto a network with symmetric interactions, where activity samples population states close to attractors. Both frameworks produce low-dimensional manifolds of population activity governed by a small number of slow variables; the latent-variable model enforces this statistically, whereas the recurrent model realizes it dynamically. Establishing the conditions under which the statistical and dynamical models of neural activity dynamics leading to robust pRG scaling are equivalent is left to future work.

### 4.3 Connections to experimental work

Our simulations represent a best-case scenario for inference. While we ran the deconvolution procedure blind to underlying ground-truth parameters, the forward model used to generate fluorescence matches the model class assumed by the deconvolution algorithm, and this is unlikely to be true for real imaged population activity. Under these ideal conditions, pairwise structure is remarkably well preserved: the Spearman correlation between cell-cell correlation matrices derived from ground-truth spikes, simulated fluorescence, and deconvolved activity is high (ρ≈0.97). Even so, eigenvalue exponents and their fit qualities shifted substantially. In real data, where indicator kinetics are heterogeneous and partially misspecified, baselines drift, shot noise and motion artifacts are present, and deconvolution priors are only approximate, we expect larger departures than those reported here.

Our model-based approach comes with two main limitations. While our fluorescence simulations were guided by paired electrophysiology-imaging data (Wei et al., 2020), scaling the model to populations of thousands of neurons required additional assumptions. We focused on a single calcium indicator (GCaMP6f-TG), but extending the forward-model approach to other indicators and modalities could reveal the conditions under which scaling relationships are most reliably measured. More broadly, similar forward-modeling approaches could be used for other high-dimensional recording modalities for which pRG analysis may provide useful insight, such as fMRI. A second limitation of our model is that it could not be used to examine how differences in the geometry of sampling across modalities may influence coarse-graining. Imaging can more easily access volumes of cells, whereas multielectrode probes typically sample neurons from different cortical layers and substructures, with potentially distinct correlation statistics. Determining how geometry affects pRG analyses will require empirical investigation, for instance by sub-sampling volumetric imaging data to clarify how layer-specific or topographic organization affects apparent scaling.

The consequences of the many choices in how neural data is recorded and processed are challenging to address solely in experimental data, in which there will always be constraints in the population size and recording duration. As a testbed to examine the impact of these choices, the dynamic latent variable model is an excellent tool: it replicates pRG scaling and exponents and can be scaled up to arbitrary population size and recording duration (Morrell et al., 2021), and can be combined, as done here, with indicator-specific forward models (Wei et al., 2020) and deconvolution methods. Ultimately, an understanding of how the measurement process shapes observed scaling could lead to the development of methods to calibrate or subtract these effects. Our results indicated that some, but not all, signatures are robust to the method in which activity is measured. Thus, the present work provides both a caution and a path forward: scaling analyses remain powerful, provided the effect of transformations between neural activity and observed data are understood.

## 5 Methods

### 5.1 Dynamic latent variable model for population spiking activity.

We use the dynamic latent variable model from Morrell et al. (2021). This is a model in which each neuron is coupled to independent latent variables that determine the time evolution of the neuron activity. Each latent variable is an Orstein-Uhlenbeck random walk. The interaction between each neuron and each latent variables is drawn from a normal distribution with mean 0 and variance $N_{F}^{-1}$ as shown in Supplementary Figure 1. Aside from shared drive arising from the common set of latent variables, neurons are independent. Activity *s*_*i*_ in neuron *i* is determined by


$$\begin{array}{l} H=\eta \overset{N,N_{F}}{\underset{i,m}{\sum }}J_{im}h_{m}\left(t\right)s_{i}+\epsilon \underset{i}{\sum }s_{i} \end{array}$$ (1)

$$\begin{array}{l} P\left(s_{i}|\left\{h_{m}\left(t\right)\right\}\right)=\frac{1}{Z\left(s_{i},\left\{h_{m}\left(t\right)\right\}\right)}e^{-H}. \end{array}$$ (2)

In Equation 1, *N* is the total number of neurons, *N*_*F*_ is the number of latent fields, *h*_*m*_(*t*) represents a given latent field, *S* is the state of the neuron, and η and ϵ are parameters that control the coupling strength and bias. In Equation 2, *H* sets the probability of activity of each neuron, given the latent variables.

The model was simulated for *N* = 1024 neurons for *T* = 2·10^4^ s (2·10^6^ time steps, or 5.5 h). At the largest value of Δ = 10 s, this translates to *T*/Δ = 2000 samples in time. For pRG analysis, scaling relationships were fit on fifths of the data, and error bars are estimated as the standard deviation across fifths of the data.

### 5.2 Phenomenological renormalization group (pRG) analysis

We applied the real-space phenomenological renormalization group (pRG) procedure introduced in Meshulam et al. (2019) and subsequently used in multiple large-scale neural datasets (Morales et al., 2023; Munn et al., 2024; Morrell et al., 2021; Castro et al., 2024). The goal of pRG is to examine how statistical structure changes under systematic coarse-graining, thereby revealing scale-invariant relationships in population activity. At each step, units that share the strongest correlations are merged, producing a hierarchy of coarse-grained descriptions that preserves the dominant shared fluctuations in the population. We refer the reader to the original work for a complete description, but walk through the procedure briefly here.

On iteration *k* = 1, the pair of neurons with the highest correlation coefficient are identified and merged into a cluster. The activity of a cluster is defined as the sum of its constituent neurons' activities, normalized by the mean activity in the non-silent state so that activity remains comparable across cluster sizes. This procedure is repeated until all neurons are assigned to clusters of size *K* = 2^*k*^ = 2. On iteration *k*+1, correlations are recomputed among the clusters of size 2^*k*^, and the merging procedure is repeated to form clusters of size 2^*k*+1^. Iterating this process yields coarse-grained variables spanning scales from single neurons to large clusters.

Below, we describe the three scaling relationships analyzed in this work.

#### 5.2.1 Variance scaling and the exponent α

For each cluster size *K*, we computed the variance $\sigma_{K}^{2}$ of the coarse-grained activity. The variance-scaling exponent α was extracted as the slope of the best-fit line of $\log\sigma_{K}^{2}$ vs. log *K*. This exponent captures how shared variability grows with cluster size, with α = 1 corresponding to uncorrelated activity and α = 2 corresponding to perfectly correlated activity among all neurons in the cluster.

#### 5.2.2 Free-energy scaling and the exponent β

Following Morrell et al. (2021), we computed the free-energy scaling exponent β from the growth of −ln *P*_silence_ with cluster size *K*, where *P*_silence_ is the probability that all neurons in a cluster are inactive in a time bin. For spike-count data, inactivity is naturally defined as a zero spike count. For deconvolved calcium traces, inactivity was defined as a bin with no inferred spikes. Because the fluorescence traces do not possess a true zero-activity state, β cannot be reliably defined for the raw *dF*/*F* signals, and we therefore report β only for spike-count and deconvolved data.

#### 5.2.3 Covariance-spectrum scaling and the exponent μ

To characterize how the structure of correlations evolves under coarse-graining, we computed the covariance matrix for each cluster of size *K*, calculated its eigenvalues, and averaged the spectra across clusters. Following previous work (Meshulam et al., 2019; Morales et al., 2023; Castro et al., 2024), we plotted the eigenvalues λ(*r*) against their scaled rank *r*/*K* and estimated the exponent μ as the slope of the best-fit line of logλ(*r*) vs. log(*r*/*K*) over the range *r* = 1 to *r* = *K*/2, sampled logarithmically (e.g., at *r* = 1, 2, 4, ...*K*/2). This exponent quantifies how rapidly covariance modes decay at each coarse-grained scale.

We used two indicators of the quality of scaling. First, we computed *R*^2^ over logarithmically spaced ranks up to half of the maximum cluster size (1, 2, 4, 8, ...*K*/2). For reference, values of *R*^2^ reported for experimental data are typically larger than 0.95 (see Table S2 of Morales et al., 2023). Second, we extracted slopes μ from fits for *K* = 32, 64, 128, and 256; smaller differences across *K* indicated higher quality of scaling. Uncertainties in exponents are reported as the standard deviation across fifths of the data.

### 5.3 Simulated imaging and deconvolution

#### 5.3.1 Simulating fluorescence

We generated synthetic calcium fluorescence signals by applying the forward model of Wei et al. (2020) to the simulated spike trains. Spikes at times {*t*_*k*_} were first converted into an intracellular calcium concentration trace *c*(*t*) using a double-exponential kernel,


$$\begin{array}{l} c\left(t\right)=\underset{t>t_{k}}{\sum }\exp\left(-\frac{t-t_{k}}{\tau_{d}}\right)\left[1-\exp\left(-\frac{t-t_{k}}{\tau_{r}}\right)\right], \end{array}$$ (3)

where τ_*d*_ and τ_*r*_ are decay and rise constants. Internal noise was omitted for simplicity. The calcium concentration was then transformed into a synthetic fluorescence signal through a sigmoidal nonlinearity,


$$\begin{array}{l} \Delta F/F_{\text{synth}}\left(t\right)=\frac{F_{\max}}{1+\exp\left[-k\left(c\left(t\right)-c_{1/2}\right)\right]}+n_{e}\left(t\right) \end{array}$$ (4)

where *F*_max_ is the maximum fluorescence change, *k* controls the steepness of the nonlinearity, *c*_1/2_ is the half-activation concentration, and $n_{e}\left(t\right)\sim N\left(0,\sigma_{\text{ext}}^{2}\right)$ is additive external noise. We simulated three noise levels, σ_ext_ = 0.02, 0.2, 0.4.

Model parameters were drawn from distributions chosen to approximate those reported for GCaMP6f-TG in Wei et al. (2020) (their Figure 4B and Supplementary Figure 3A). *F*_max_ was drawn from a normal distribution with mean 8 and standard deviation 2. To avoid saturation at high firing rates, the rise and decay constants were made weakly dependent on each cell's average firing rate *r*_*i*_: τ_*r*_ was drawn from a lognormal distribution in which logτ_*r*_ had mean log(0.05)+0.2log(*r*_*i*_) (s.d. 0.3), and τ_*d*_ was set to τ_*r*_+*c*_0_/(τ_0_+*r*_*i*_), with *c*_0_ = 0.5 and τ_0_ = 0.4. For each neuron, *c*_1/2_ was set to half of the maximum calcium concentration. Because *c*_1/2_ and *k* are tightly coupled, we used the empirical relationship


$$\begin{array}{l} c_{1/2}=\frac{32}{7k+1}, \end{array}$$ (5)

which was obtained from the parameters extracted from Wei et al. (2020). This relationship should be viewed as a heuristic.

Fluorescence signals were then sampled at 10 Hz (Δ = 100 ms), matching a typical imaging frame rate. In all pRG analyses below, fluorescence traces were evaluated at this native temporal resolution.

#### 5.3.2 Deconvolution of simulated fluorescence

Fluorescence traces were deconvolved using the constrained foopsi algorithm (Giovannucci et al., 2019; Pnevmatikakis et al., 2016) with the “lars” optimization method, as implemented in the software package of Wei (2020). For each neuron, the baseline fluorescence was initialized as the median of the full fluorescence trace, and baseline drift was not modeled. Calcium decay time constants were estimated by fitting AR(2) models to ten nonoverlapping 100 s segments of the fluorescence trace, and the slow pole of the AR model was taken as the estimate of τ_*d*_. In cases where no stable AR(2) model was found, we increased model order up to 5; if no stable solution was obtained, deconvolution for that neuron was excluded. This occurred most frequently at higher levels of noise, and accounts for the missing fraction of cells in Figure 3G.

Estimated decay constants were used to initialize the full deconvolution, which was run in batches (batch size 2000) over the full 200,000-frame dataset. The output was an inferred spike train for each neuron, which we used for subsequent pRG analysis.

#### 5.3.3 Correlation analysis

Because the pRG procedure relies on the ordering of pairwise correlations to form clusters, we computed correlation matrices for spike counts, simulated fluorescence, and deconvolved traces at the same temporal resolution (Δ = 100 ms). For each noise condition, we computed Pearson correlations across time for all neuron pairs, as well as the Spearman correlation between the ranking of spike-count correlations and the rankings obtained for fluorescence and deconvolved signals. These analyses verified that pairwise correlations were highly preserved across observational transformations, providing a consistent substrate for pRG coarse-graining.

## Data Availability

The original contributions presented in the study are included in the article/Supplementary material, further inquiries can be directed to the corresponding author.
