# Supplementary material for: Measurement effects on critical scaling in neural systems
Source: Front Comput Neurosci. 2026 Jan 23;19:1724190. doi: 10.3389/fncom.2025.1724190 (PMC12876168; doi:10.3389/fncom.2025.1724190)
Supplement: Supplementary file 1 [file Data_Sheet_1.pdf]

Supplementary Material

1 SUPPLEMENTARY DATA

Supplementary Material is provided as a separate PDF file and includes additional figures supporting the main text. All supplementary files will be deposited to FigShare for permanent storage and assigned a DOI by the publisher.

2 SUPPLEMENTARY TABLES AND FIGURES

2.1 Figures

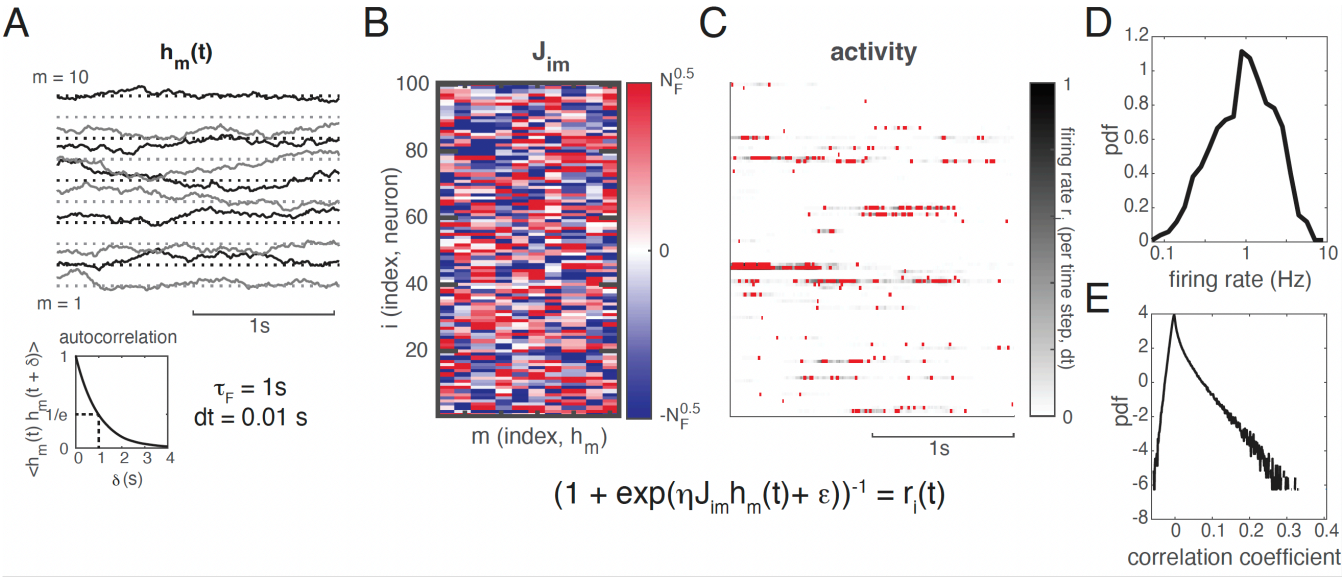

Figure S1. Simulation of large neural populations with realistic firing rates and correlation distributions using a dynamic latent variable model. **(A)** Evolution over of a set of 10 latent variables. *inset, below:* Autocorrelation of the latent variables. **(B)** Coupling matrix $J_{im} \sim N(0, N_F^{-1})$ determines relationship between each latent variable m and each neuron i. **(C)** Neural activity rate r_i (black and white gradient) with the simulated spiking activity overlaid (red ticks). **(D)** Distribution of the firing rates across neurons in the network for parameters used in this study. **(E)** Distribution of correlation coefficients generated by the model.

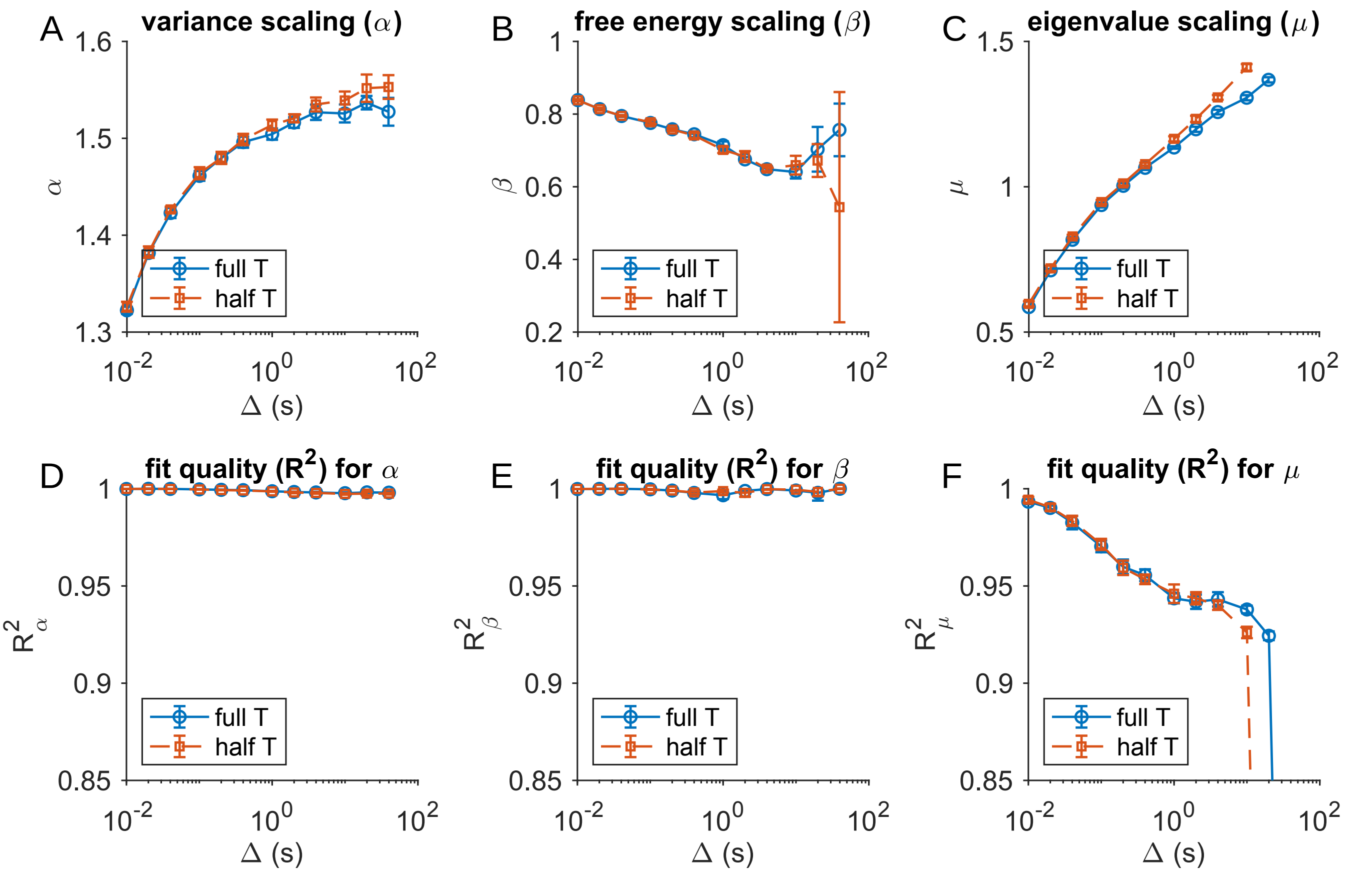

Figure S2. Finite-size robustness of pRG scaling exponents and fit quality. (A–C) Comparison of scaling exponents α, β, and μ obtained from the full-length simulated dataset (solid circles) and from a dataset of half the duration (dashed squares). Each point reflects the mean exponent estimated across splits of the full dataset at a given temporal bin size Δ; error bars denote the standard deviation across splits. Full simulation was $T = 20000$ s and five splits were used, so for "full T" each split is 4000 s and for "half T" each split is 2000 s. The exponent μ is computed from the eigenvalue–rank relation using the largest cluster size K, consistent with the main-text analyses. Across all exponents, the half-length dataset reproduces the full-length dependence on Δ, indicating that the scaling relationships and their degradation at large Δ do not arise from finite-sample limitations of the simulated time series. **(D–F)** Corresponding fit quality (R^2) for the α, β, and μ fits, averaged across windows and plotted as a function of Δ. Error bars denote the standard deviation of R^2 across data splits. For μ, the R^2 values shown correspond to the same cluster size used in panel C. Fit quality remains high across bin sizes and is nearly identical for full- and half-duration data, further confirming that the trends observed in the exponents are not driven by recording length. The greater variability in R^2 for μ reflects the higher sensitivity of eigenvalue-rank fits to finite-size effects and observational transformations, as described in the main text.
